# Mechanism of transcription-coupled DNA modification recognition

**DOI:** 10.1186/s13578-016-0133-3

**Published:** 2017-02-22

**Authors:** Ji Hyun Shin, Liang Xu, Dong Wang

**Affiliations:** Department of Cellular and Molecular Medicine, School of Medicine, Skaggs School of Pharmacy and Pharmaceutical Sciences, University of California San Diego, La Jolla, CA 92093 USA

**Keywords:** Transcription, RNA polymerase II, DNA damage, Transcription-coupled repair, Transcriptional pausing, Transcriptional arrest, Transcriptional lesion bypass, UV DNA damage, Oxidative DNA lesions, 8-Oxo-2′-deoxyguanosine

## Abstract

As a key enzyme for gene expression, RNA polymerase II (pol II) reads along the DNA template and catalyzes accurate mRNA synthesis during transcription. On the other hand, genomic DNA is under constant attack by endogenous and environmental stresses. These attack cause many DNA lesions. Pol II functions as a specific sensor that is able to recognize changes in DNA sequences and structures and induces different outcomes. A critical question in the field is how Pol II recognizes and senses these DNA modifications or lesions. Recent studies provided new insights into understanding this critical question. In this mini-review, we would like to focus on three classes of DNA lesions/modifications: (1) Bulky, DNA-distorting lesions that block pol II transcription, (2) small DNA lesions that promote pol II pausing and error-prone transcriptional bypass, and (3) endogenous enzyme-catalyzed DNA modifications that lead to pol II pausing and error-free transcriptional bypass.

## Background

RNA polymerase II (pol II) is the enzyme responsible for the transcription and synthesis of pre-messenger RNA and noncoding RNA transcripts [1]. During the process of transcription, pol II reads along the template strand of genomic DNA and incorporates the matched nucleotide substrate with high fidelity to ensure accurate genetic transfer and minimize transcriptional errors. Transcriptional fidelity during elongation is maintained via at least three fidelity checkpoint steps: the nucleotide insertion step, RNA transcript extension step, and proofreading step [1]. Unavoidably, pol II may encounter various DNA modifications or lesions during its long transcriptional ‘journey’ moving along the DNA template. In such situations, pol II utilizes several important motifs to ‘sense’ these DNA modifications. The distinct interactions between pol II conserved motifs and these DNA modifications also induce appropriate transcription-coupled responses, which may lead to transcriptional mutagenesis, transcription-coupled repair pathway, or apoptosis [2–4].

## Main text

**Fig. 1:**
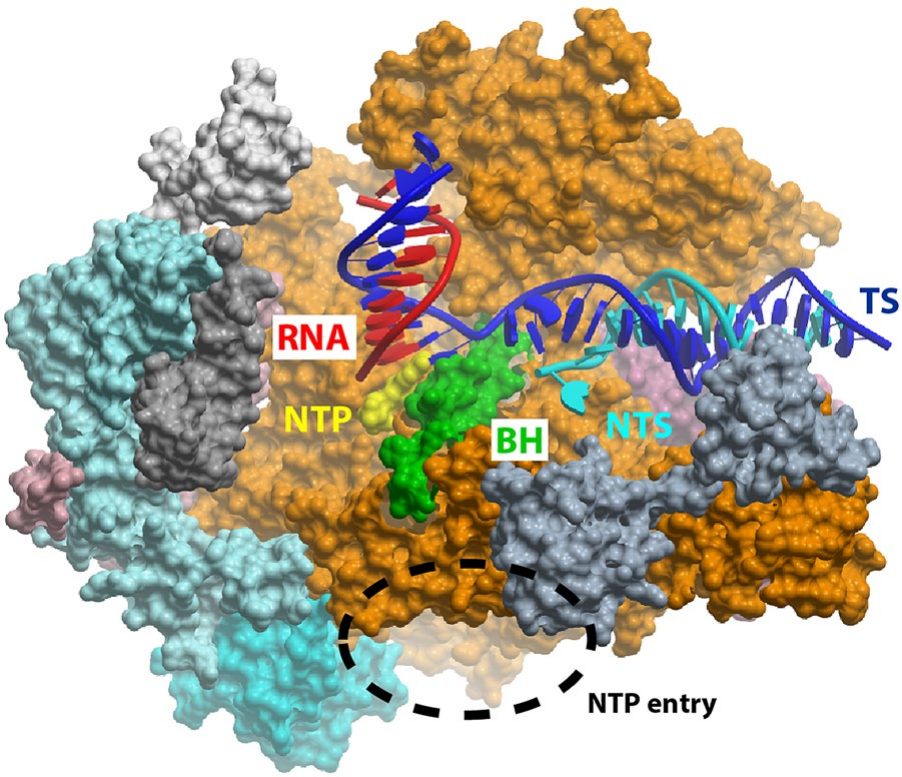
Structure of RNA polymerase II elongation complex. The incoming *NTP* enters the pol II active site through the secondary channel of pol II (*dashed circle*). The bridge helix (*BH*) is shown in *green*, while the RNA, template DNA (*TS*), and non-template DNA (*NTS*) are shown in *red*, *blue*, and *cyan*, respectively

There are several important conserved structural components of pol II involved in DNA template base recognition and fidelity control, including the trigger loop and bridge helix of the Rbp1 subunit (Fig. 1). The trigger loop (TL) is a highly conserved domain in various multisubunit RNA polymerases that is responsible for the rapid catalysis of phosphodiester bond formation and maintaining substrate specificity [1, 5, 6]. In the presence of a matched NTP substrate, complementary to the DNA template in the active site, the TL undergoes a conformational change from open, inactive states to a closed, active state and positions the substrate for catalysis. The bridge helix is a long alpha helix domain that bridges over the two halves of pol II and separates the pol II catalytic site from the downstream main channel and the secondary channel [5, 7, 8]. All of these components are important for pol II enzymatic activity, but they also contribute to the ability of pol II to sense DNA modifications and damage during transcription elongation.

**Fig. 2:**
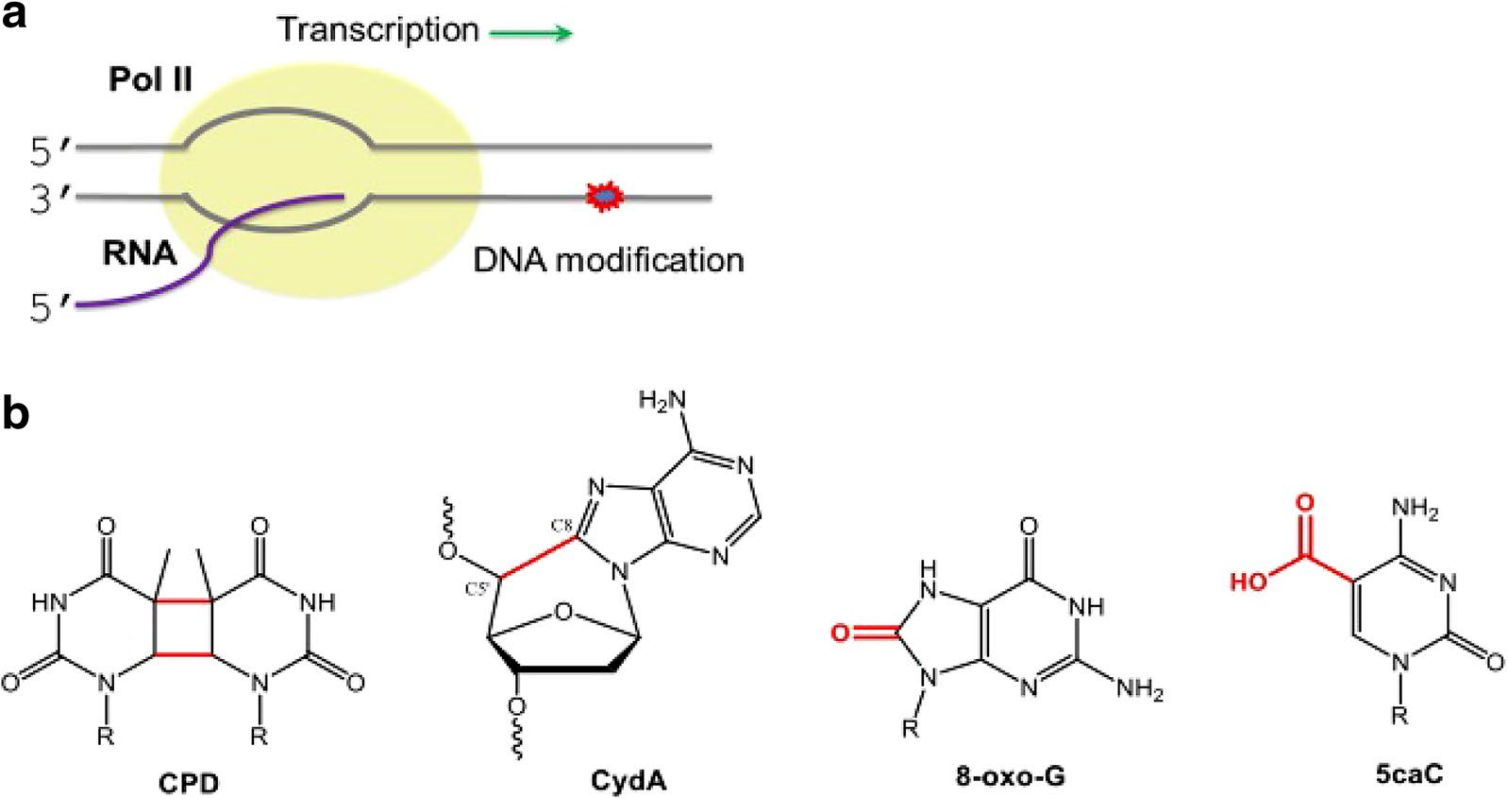
**a** Elongation of RNA polymerase II may encounter different types of DNA modifications. **b** These include bulky, DNA-distorting lesions (e.g. UV-induced cis-syn CPD, oxidative damage CydA), small but mutagenic DNA damage (e.g. 8-oxo-guanine), and enzyme-catalyzed endogenous DNA modifications (e.g. 5caC)

Genomic DNA is under constant attack, including endogenous reactive oxygen species and free radicals, and external factors like UV irradiation. As a result, these attacks cause many DNA lesions, including base modifications, strand breaks, crosslinks, and bulky, DNA-distorting lesions. Pol II may encounter these lesions or modifications during RNA transcript synthesis (Fig. 2). A critical question in the field is how Pol II recognizes and senses these DNA modifications or lesions. Recent studies provided new insights into understanding this critical question. In this mini-review, we would like to focus on three classes of DNA lesions/modifications: (1) Bulky, DNA-distorting lesions that block pol II transcription, (2) small DNA lesions that promote pol II pausing and error-prone transcriptional bypass, and (3) endogenous enzyme-catalyzed DNA modifications that lead to pol II pausing and error-free transcriptional bypass.

**Fig. 3:**
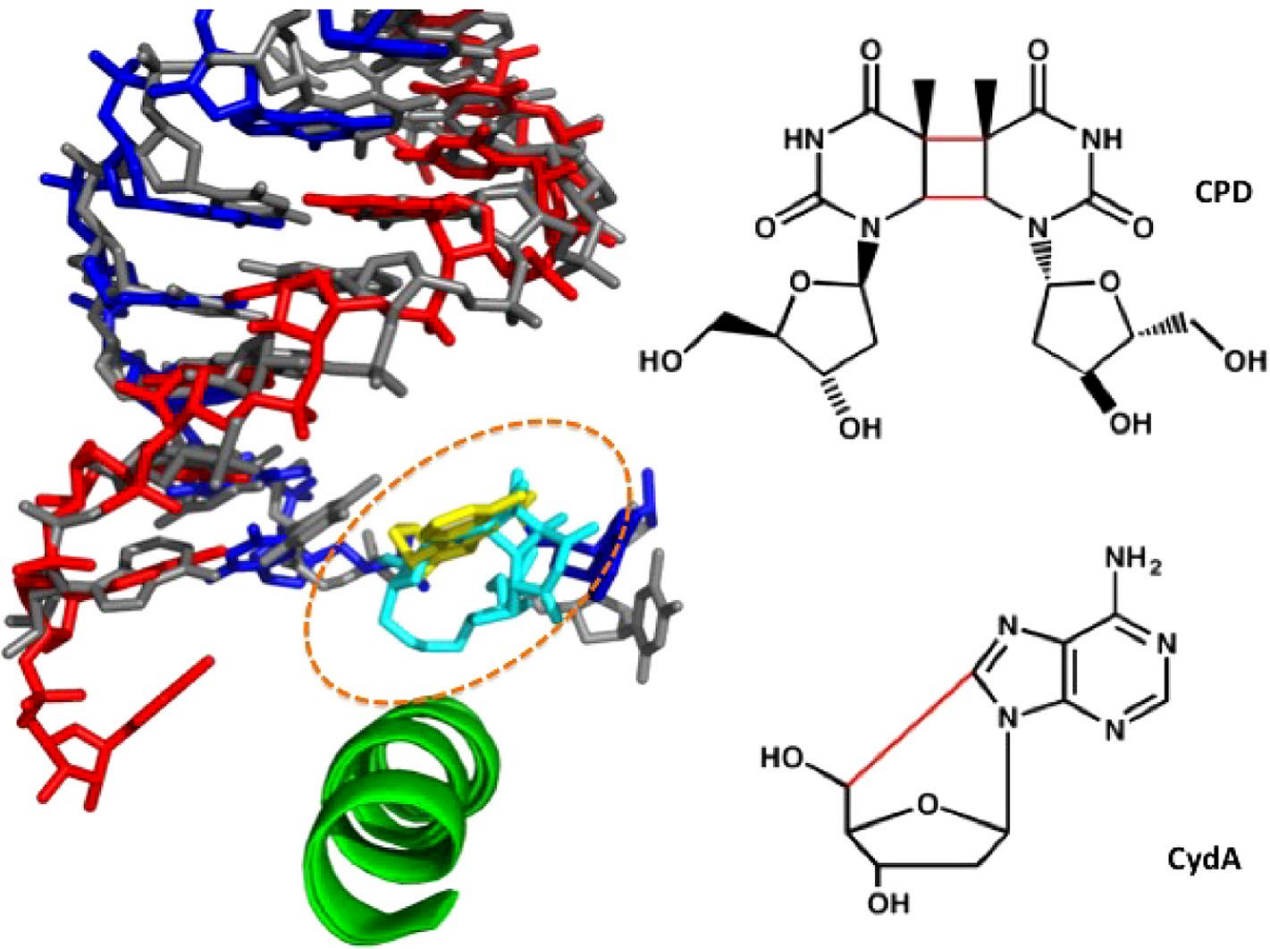
Structural overlay of RNA pol II elongation complexes that accommodates cis-syn CPD or CydA lesion at the “above-bridge-helix” conformation (*dashed circle*) and causes transcriptional arrest. The bridge helix is shown in *green*, and RNA and DNA are shown in *red* and *blue*, respectively

Bulky DNA-distorting lesions serve as a strong road block for pol II elongation [9]. UV-induced cyclobutane pyrimidine dimer (CPD) lesions form 1,2-intrastrand cross-links that significantly distort the DNA template structure. These lesions strongly inhibit pol II transcription by reducing the rate and fidelity of substrate incorporation and extension [10, 11]. Intriguingly, a structurally unrelated bulky DNA lesion, cyclopurines (CydA), which arise form oxidative damage, also strongly inhibit pol II transcription elongation in the similar manner [12, 13]. In both cases of transcriptional stalling, pol II utilizes the A rule, a phenomenon in which nucleotide incorporated in a slow, error-prone, and non-template dependent manner (AMP is preferentially incorporated regardless the template), opposite a damaged DNA base [11, 13], indicating that pol II may recognize these structurally different DNA lesions in a similar manner. Intriguingly, further structural analysis indeed revealed that both lesions are accommodated above the bridge helix (Fig. 3) and arrested in a similar position in which the damaged base is stuck at the half-way position of template translocation between the i+1 and the i+2 position [11, 13]. Interestingly, such DNA damage induced translocation-arrested states were very similar to the transient translocation intermediate states of normal pol II translocation of a non-damaged DNA template observed by molecular dynamic simulation [14]. These translocation intermediate states were proposed to be rate-limiting steps during normal translocation, as they require significant conformational changes for the DNA template base to crossover the bridge helix to progress through the active site [14]. Therefore, the presence of bulky DNA lesions introduces a great steric barrier to the crossover of the bridge helix and causes pol II arrest at this ‘half-way’ translocation state. These common lesion arrest mechanisms indicate that the rate-limiting bridge helix crossover step acts as a critical checkpoint for pol II to examine the DNA template and recognize bulky DNA lesions that greatly compromise DNA backbone flexibility and integrity.

Some small DNA lesions do not affect the DNA backbone significantly and therefore do not block transcription elongation. Rather, some of these DNA lesions cause error-prone transcriptional lesion bypass. For example, 8-Oxo-2′-deoxyguanosine (8-oxo-dG), a common endogenous oxidative damage, is one such mutagenic DNA lesion [15]. Pol II can either insert a matched cytosine or a mismatched adenine when it encounters 8-oxo-dG during transcription [16, 17]. However, the presence of the 8-carbonyl group of 8-oxo-dG destabilizes the canonical *anti* conformation of template base, making ATP misinsertion and extension much more energy favorable [17]. Consequently, the presence of 8-oxoG at the DNA template causes a specific C→A mutation in the RNA transcript, termed transcriptional mutagenesis [18]. Emerging evidence suggests that transcriptional mutagenesis could contribute to cancer, aging, and a variety of neurodegenerative diseases.

**Fig. 4:**
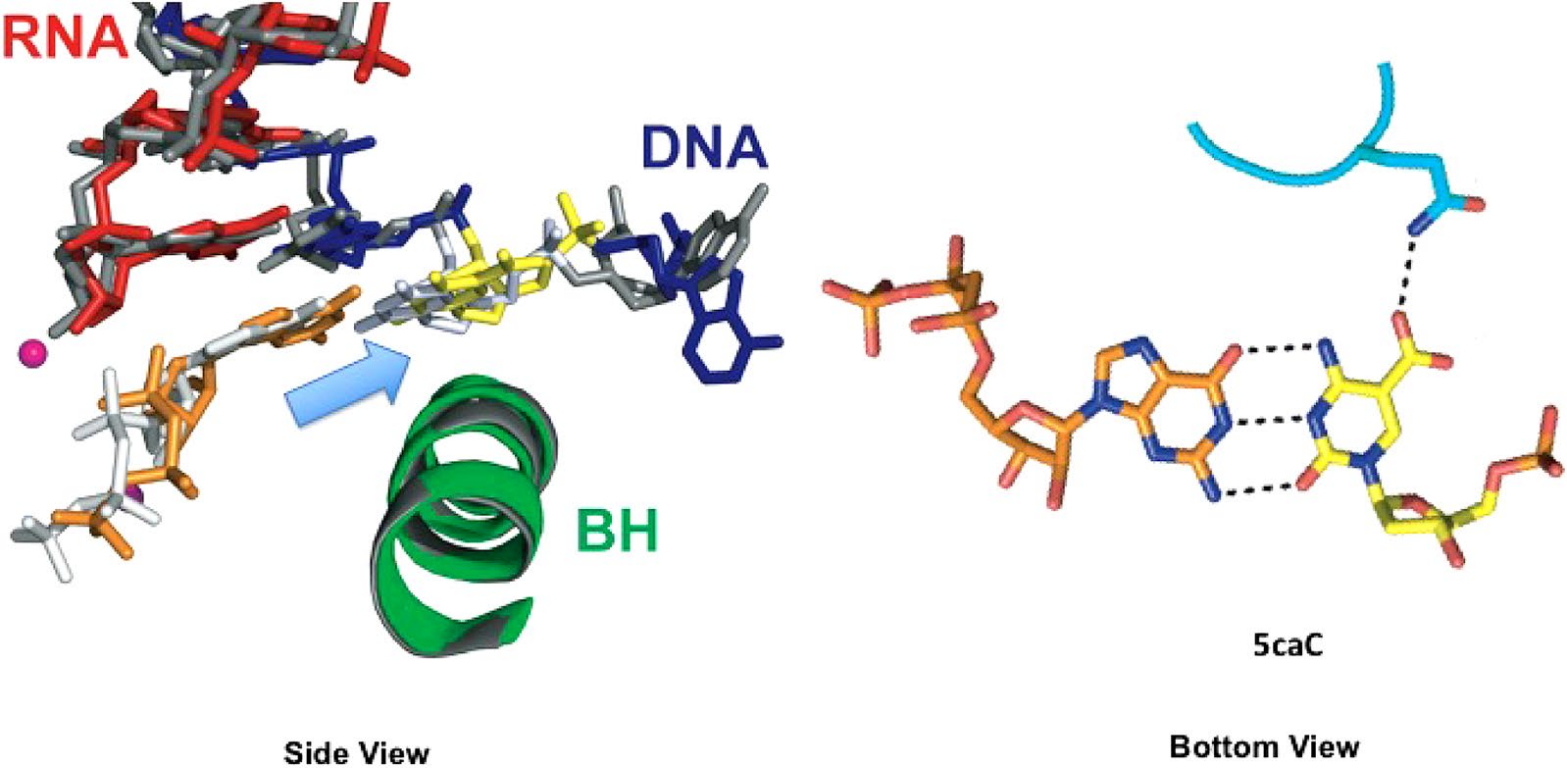
The structure of RNA pol II elongation complex with 5caC, in which 5caC adopts the similar “above-bridge-helix” conformation. 5caC can form a specific hydrogen bond with key residue Q531 of the Rpb2 subunit. The bridge helix is shown in *green*, and RNA and DNA are shown in *red* and *blue*, respectively

The third class of DNA modifications are generated by endogenous enzymes. For example, the methylation of cytosine to 5-methylcytosine (5mC) by DNA methyltransferases (DNMTs) is the most common epigenetic DNA modification, often enriched at enhancer and promoter regions. 5mC functions as an epigenetic mark and plays an important role in regulating gene transcription and chromatin structure [19]. On the other hand, 5mC can also undergo active demethylation, a process catalyzed by ten eleven translocation (Tet) proteins to generate the oxidized mC (oxi-mC) intermediates, 5-hydroxymethylcytosine (5hmC), 5-formylcytosine (5fC), and 5-carboxylcytosine (5caC), before being removed by thymine DNA glycosylase (TDG) to regenerate the unmodified cytosine [20]. Recent evidence suggests that 5fC and 5caC are not merely reaction intermediates, but also play novel functional roles in gene regulation, as they are able to recruit various transcription factors and DNA repair protein complexes, as well as to induce transient pausing of pol II in vitro [21, 22]. Recently, structural studies revealed that pol II interacts with 5caC via specific interactions between pol II and the 5caC. These specific interactions drag the majority of 5caC to be accommodated above the bridge helix (Fig. 4). Further structural analysis revealed that a conserved ‘epi-DNA recognition loop’, located in the fork region of the Rpb2 subunit of pol II, is responsible for the recognition of 5caC in the major groove of the template strand (Fig. 4) [23]. Notably, the presence of 5caC can still support Watson–Crick base pair with incoming GTP substrate. However, the specific hydrogen bonds between the epi-DNA recognition loop and 5caC disrupts proper alignment of the substrate and 3′-RNA terminus, and results in a partially open conformation of the trigger loop [23]. Without full closure of the trigger loop, GTP addition efficiency is significantly reduced. The Q531A mutant abolishes the ability of epi-DNA recognition loop to form the hydrogen bond with 5caC and consequently gained a significant increase in GTP incorporation specificity. Conclusively, the evidence showed that the specific hydrogen bonding between Q531 of pol II and the carboxyl group of 5caC causes a positional shift of the incoming GTP and compromises nucleotide addition, resulting in the significant reduction of pol II elongation.

Taken together, the different mechanisms of pol II arrest or bypass of a variety of lesions or modifications support the idea that pol II is a specific sensor that detects DNA modifications during transcription. The specific interactions between DNA lesions/modifications and pol II govern the specific transcriptional outcomes: transcriptional arrest, pausing, and error-prone or error-free transcriptional lesion bypass. For bulky, DNA-distorting lesions such as cis-syn CPD and CydA lesions, the presence of DNA lesions compromises DNA backbone flexibility and greatly slows down the bridge helix crossover step during translocation, thus forming a strong road block for pol II transcription elongation [1]. This DNA-lesion induced pol II arrest initiates transcription-coupled nucleotide excision repair [2]. For the 8-oxo-dG lesion, the interaction between the 8-oxo-dG and the active site of pol II promotes the mis-incorporation of an adenine base opposite the lesion and leads to error-prone transcriptional bypass. 8-oxo-dG is a common type of oxidative DNA damage and can be effectively repaired by the base excision repair pathway. Whether 8-oxo-dG is subject to transcription-coupled repair has been an interesting debatable topic for decades, but emerging new evidence suggests that 8-oxoG is preferentially repaired in the transcribed strand in vivo, yet the detailed molecular mechanism remains to be established [24]. With regards to the enzyme-catalyzed 5caC modifications, RNA pol II can directly sense the 5caC modification via the specific interaction between pol II and 5caC [23]. This 5caC-induced transcriptional pausing may suggest another layer of functional interplay between epigenetic DNA modifications and pol II transcription machinery in the fine-tuning of transcriptional dynamics and gene expression [25, 26].

## Conclusion

Conclusively, RNA polymerase II can sense a variety of different DNA structures/lesions during transcription and induce specific transcription-coupled responses including transcriptional lesion bypass, transcriptional pausing and arrest, which may consequently trigger DNA repair or cell death. As RNA pol II scans along significant portions of the genomic DNA during transcription, the sensory function of pol II possibly may have developed as an evolutionary mechanism for the cell to maintain genomic integrity, to respond a variety of environmental cues or stress, and to determine how and when the cell’s energy and resources should be optimally utilized.

## Abbreviations

pol II: RNA polymerase II
TL: trigger loop
5mC: 5-methylcytosine
Tet: ten eleven translocation proteins
oxi-mCs: oxidized methylcytosines
5hmC: 5-hydroxymethylcytosine
5fC: 5-formylcytosine
5caC: 5-carboxylcytosine
TDG: thymine DNA glycosylase
CPD: cyclobutane pyrimidine dimer lesions
CydA: cyclopurines
8-oxo-dG: 8-Oxo-2′-deoxyguanosine
